# Genetic and Cellular Interaction During Cardiovascular Development Implicated in Congenital Heart Diseases

**DOI:** 10.3389/fcvm.2021.653244

**Published:** 2021-03-16

**Authors:** Kazuki Kodo, Keiko Uchida, Hiroyuki Yamagishi

**Affiliations:** Division of Pediatric Cardiology, Department of Pediatrics, Keio University School of Medicine, Tokyo, Japan

**Keywords:** heart field, neural crest, outflow tract, inositol trisphosphate receptor, TBX1, 22q11.2 deletion

## Abstract

Congenital heart disease (CHD) is the most common life-threatening congenital anomaly. CHD occurs due to defects in cardiovascular development, and the majority of CHDs are caused by a multifactorial inheritance mechanism, which refers to the interaction between genetic and environmental factors. During embryogenesis, the cardiovascular system is derived from at least four distinct cell lineages: the first heart field, second heart field, cardiac neural crest, and proepicardial organ. Understanding the genes involved in each lineage is essential to uncover the genomic architecture of CHD. Therefore, we provide an overview of recent research progress using animal models and mutation analyses to better understand the molecular mechanisms and pathways linking cardiovascular development and CHD. For example, we highlight our recent work on genes encoding three isoforms of inositol 1,4,5-trisphosphate receptors (IP_3_R1, 2, and 3) that regulate various vital and developmental processes, which have genetic redundancy during cardiovascular development. Specifically, IP_3_R1 and 2 have redundant roles in the atrioventricular cushion derived from the first heart field lineage, whereas IP_3_R1 and 3 exhibit redundancy in the right ventricle and the outflow tract derived from the second heart field lineage, respectively. Moreover, 22q11.2 deletion syndrome (22q11DS) is highly associated with CHD involving the outflow tract, characterized by defects of the cardiac neural crest lineage. However, our studies have shown that *TBX1*, a major genetic determinant of 22q11DS, was not expressed in the cardiac neural crest but rather in the second heart field, suggesting the importance of the cellular interaction between the cardiac neural crest and the second heart field. Comprehensive genetic analysis using the Japanese genome bank of CHD and mouse models revealed that a molecular regulatory network involving GATA6, FOXC1/2, TBX1, SEMA3C, and FGF8 was essential for reciprocal signaling between the cardiac neural crest and the second heart field during cardiovascular development. Elucidation of the genomic architecture of CHD using induced pluripotent stem cells and next-generation sequencing technology, in addition to genetically modified animal models and human mutation analyses, would facilitate the development of regenerative medicine and/or preventive medicine for CHD in the near future.

## Introduction

Congenital heart disease (CHD) is the most common life-threatening congenital anomaly that occurs in ~1% of live births. With advances in pediatric cardiology and cardiac surgery, most patients with CHD survive to adulthood; therefore, understanding the inheritance of CHD has become an increasingly critical clinical issue. Although insight gleaned from molecular genetics combined with developmental biology approaches has helped to uncover the detailed mechanisms of cardiovascular development, the genomic architecture of CHD remains largely unknown. We provide an overview of the progress that research has made till date in understanding the molecular mechanisms contributing to cardiovascular development, which in turn can provide new directions for research to uncover the inheritance of CHD and key susceptibility genes. We first provide general background into the etiology of CHD and the nature of cardiac development, highlighting our work on the role of inositol 1,4,5-trisphosphate receptors (IP_3_Rs) in this process. In addition, we focus on recent research demonstrating a mechanistic link of the T-box-containing transcription factor (TBX1) with CHD in the context of 22q11.2 deletion syndrome (22q11DS). Finally, we highlight progress to date in understanding the general genetic architecture associated with CHD and the underlying regulatory mechanisms.

## Etiology of CHD

CHD is considered to occur due to defects in cardiovascular development during the first 6 weeks of gestation. At this stage, the heart and vessels develop from a simple primitive tube structure into a four-chambered heart with two great vessels. Genetic factors, including chromosomal abnormalities, are estimated to account for approximately 8% of CHD cases, with single-gene mutations accounting for about 5% of cases, and environmental factors, including maternal infections, systemic diseases, and administration of drugs, accounting for about 2% of CHD cases. However, the etiology of the remaining ~85% of CHDs is generally unknown, and is therefore attributed to so-called “multifactorial inheritance,” which refers to the interaction between certain genetic and environmental factors (1, 2). Recently, more genetic factors associated with CHD have been reported, including chromosomal abnormalities for 12% of cases, *de novo* copy number variants such as chromosomal microdeletion accounting for 15% of cases and *de novo* gene mutation affecting protein function in 10% of cases, and inherited gene mutations in 1.3% of cases (Tables 1, 2) (3–5). As shown in Tables 1, 2, candidate monogenic factors include many transcription factors and signal molecules that are essential for development of the heart and are responsible for multiple types of CHD. Genetic alterations of these factors are considered to disrupt the spatiotemporal regulation of complex three-dimensional heart structure. However, the interaction of multiple genetic and environmental factors is still considered as the primary etiology of the remaining majority of CHDs.

**Table 1:** Genetic causes of non-syndromic congenital heart diseases.

	**Gene**	**Cardiovascular malformation**	**Gene MIM**
Transcription factors	*CITED2*	ASD, VSD, AS, PS, SIT, Dextrocardia, TGA, TOF, RVOTO, TAPVR	602937
	*GATA4*	Dextrocardia, AVSD, DORV,TOF, BAV, CoA, AR, PAPVR,PDA, PS, ASD, VSD	600576
	*GATA5*	AVSD, DORV, LVNC, BAV, CoA	611496
	*GATA6*	AVSD, TOF, PDA, PTA, PS, ASD, VSD	601656
	*MED13L*	TGA	608771
	*NR2F2*	AVSD, AS, CoA, VSD, HLHS, TOF, DORV	107773
	*NKX2–5*	ASD, AVSD, BAV, CoA, Dextrocardia, DORV, Ebstein's anomaly, HTX, HLHS, IAA, LVNC, Mitral valve anomalies, PA, PAPVR, PDA, PS, SVAS, TA, TAPVR, TGA, TOF, PTA, VSD	600584
	*NKX2–6*	PTA	611770
	*TBX1*	DORV, TOF, IAA, PTA, VSD,	602054
	*TBX2*	ASD, VSD, RVOTO	600747
	*TBX5*	AVSD, TOF, BAV, CoA, ASD, VSD	601620
	*TBX20*	ASD, VSD, MS, DCM, LVNC	606061
	*MEF2C*	DORV	600602
	*ZFPM2/FOG2*	AVSD, DORV, TOF, VSD	603693
	*FOXH1*	TOF, TGA, HTX, VSD	603621
	*FOXO1*	TOF	136533
	*FOXP1*	AVSD, HLHS	605515
	*HAND1*	AVSD, DORV, HLHS, HLV, HRV, ASD, VSD	602406
	*HAND2*	TOF, LVNC, VSD	602407
	*MSX1*	BAV, CoA	142983
	*NFATC1*	TOF, LVNC, BAV, CoA, TA, VSD	600489
	*ETS1*	DORV, HLHS, ASD, VSD	164720
	*JARID2*	Left-sided lesions	601594
	*NR1D2*	AVSD	602304
	*RBPJ*	HLHS	147183
	*RFX3*	PTA	601337
Cell signaling and adhesion proteins	*ACVR1/ALK2*	HTX, AVSD, DORV, TGA, Left-sided lesions, ASD	102576
	*ACVR2B*	HTX, Dextrocardia, AVSD, DORV, TGA, HLHS, PS, Venous anomaly	602730
	*BMPR1A*	AVSD	601299
	*BMPR2*	AVSD, PDA, PAPVR, ASD, VSD	600799
	*GDF1*	HTX, AVSD, DORV, TGA, TOF	602880
	*SMAD6*	HLHS, AS, BAV, CoA	602931
	*CRELD1*	ASD, AVSD	607170
	*GJA1*	HLHS, VSD, PA	121014
	*JAG1*	Aortic dextroposition, TOF, BAV, CoA, PS, VSD	601920
	*NOTCH1*	HTX, AVSD, TOF, HLHS, LVNC, BAV, CoA, AS, MS, VSD	190198
	*NOTCH2*	AVSD, TOF, BAV, CoA, PS,	600275
	*PDGFRA*	TAPVR	173490
	*TAB2*	BAV, AS, TOF	605101
	*ADAM17*	AVSD	603639
	*HES1*	TGA	139605
	*HEY2*	AVSD	604674
	*APC*	BAV, CoA	611731
	*DCHS1*	LVNC, MVP	603057
	*DVL1*	LVNC, PDA	601365
	*EDN1*	TOF	131240
	*PCDHA9*	HLHS	606315
	*VEGFA*	TOF, PDA, PTA, AS, BAV, CoA, IAA, VSD	192240
Structural proteins	*ACTC1*	ASD, HCM, DCM, LVNC	102540
	*DCHS1*	MVP	603057
	*ELN*	SVAS	130160
	*MYH6*	ASD, HCM, DCM	160710
	*MYH7*	Ebstein's anomaly, LVNC, HCM, DCM	160760
	*MYH11*	PDA, TAA	160745

*AS, aortic stenosis; ASD, atrial septal defect; AVSD, atrioventricular septal defect; BAV, bicuspid aortic valve; CoA, coarctation of the aorta; DCM, dilated cardiomyopathy; DORV, double outlet right ventricle; HCM, hypertrophic cardiomyopathy; HLHS, hypoplastic left heart syndrome; HLV, hypoplastic left ventricle; HRV, hypoplastic right ventricle; HTX, heterotaxy; IAA, interrupted aortic arch; LVNC, left ventricular noncompaction; MS, mitral stenosis; MVP, mitral valve prolapse; PA, pulmonary atresia; PAPVR, partial anomalous pulmonary venous return; PDA, patent ductus arteriosus; PS, pulmonary valve stenosis; PTA, persistent truncus arteriosus; RVOTO, right ventricular outflow tract obstruction; SIT, situs inversus totalis; SVAS, supravalvular aortic stenosis; TA, tricuspid atresia; TAA, thoracic aortic aneurysm; TAPVR, total anomalous pulmonary venous return; TGA, transposition of the great arteries; TOF, tetralogy of Fallot; VSD, ventricular septal defect*.

**Table 2:** CNVs associated with CHD.

**Locus**	**Size (kb)**	**Mode**	**CNV**	**Copy number**	**Candidate genes for CHD**	**Type of CHD**
1q21.1	418-3,981	*de novo*, inherited, n/a	Gain, Loss	3–45	*PRKAB2, FM05, CHD1L, BCL9, ACP6, GJA5, CD160, PDZK1, NBPF11, JM05, GJA8*	TOF, AS, COA, PA, VSD
3p25.1	175-12,380	*de novo*, inherited	Gain	2	*RAFJ, TMEM40*	TOF
3q22.1-3q26.1	680-32134	inherited, n/a	Gain, Loss	0–300	*FOXL2, NPHP3, FAM62C, CEP70, FAIM, PIK3CB, FOXL2, BPESC1*	DORV, TAPVR, AVSD
4q22.1	45	*de novo*	Gain	1	*PPM1K*	TOF
5q14.1-q14.3	4937-5454	Inherited, *de novo*	Gain	41,103	*EDIL3, VCAN, SSBP2, TMEM167A*	TOF
5q11.1	0.6	*de novo*	Gain	1	*ISL1*	TOF
5q35.3	264-1777	*de novo*, n/a	Gain	19–38	*CNOT6, GFPT2, FLT4, ZNF879, ZNF 345C, ADAMTS2, NSD1*	TOF
7q11.23	330-348	n/a	Gain	5–8	*FKBP6*	HLHS, Ebstein
8p23.1	67-12,000	n/a	Gain, Loss	4	*GATA4,NEIL2, FDFT1, CSTB, SOX7*	AVSD, VSD, TOF, ASD, BAV
9q34.3	190-263	*de novo*	Loss	2–9	*NOTCH1, EHMT1*	TOF, COA, HLHS
9q34.3	1.7	*de novo*	Gain	1	*NOTCH1*	TOF
11p15.5	256-271	n/a	Gain	13	*HRAS*	SV, AS
13q14.11	55-1430	n/a, *de novo*	Gain	7	*TNFSF11*	TOF, TAPVR, VSD, BAV
15q11.2	238-2,285	n/a	Loss	4	*TUBGCP5, CYFIP1, NIPA2, NIPA1*	COA, ASD, VSD, TAPVR
16p13.11	1414-2903	n/a	Gain	11–14	*MYH11*	HLHS
18q11.1-2	308-6118	n/a	Gain	1–28	*GATA6*	VSD
19p13.3	52-805	n/a, *de novo*	Gain, Loss	1–34	*MIER2, CNN2, FSTL3, PTBP1, WDR18, GNA11, S1PR4*	TOF
22q11.21	0.7-13	*de novo*	Gain	1	*PRODH*	TOF
Xp22.2	509-615	n/a	Gain	2–4	*MID1*	TOF, AVSD

*CNV, copy number variation; CHD, congenital heart disease; ASD, atrial septal defect; VSD, ventricular septal defect; PDA, patent ductus arteriosus; TOF, tetralogy of Fallot; COA, coarctation of the aorta; TAPVR, total anomalous pulmonary venous return; AVSD, atrioventricular septal defect; PA, pulmonary atresia; DORV, double outlet right ventricle; BAV, bicuspid aortic valve; HLHS, hypoplastic left heart syndrome; AS, aortic stenosis; SV, single ventricle*.

## Developmental Origins of the Cardiovascular System

**Figure 1:**
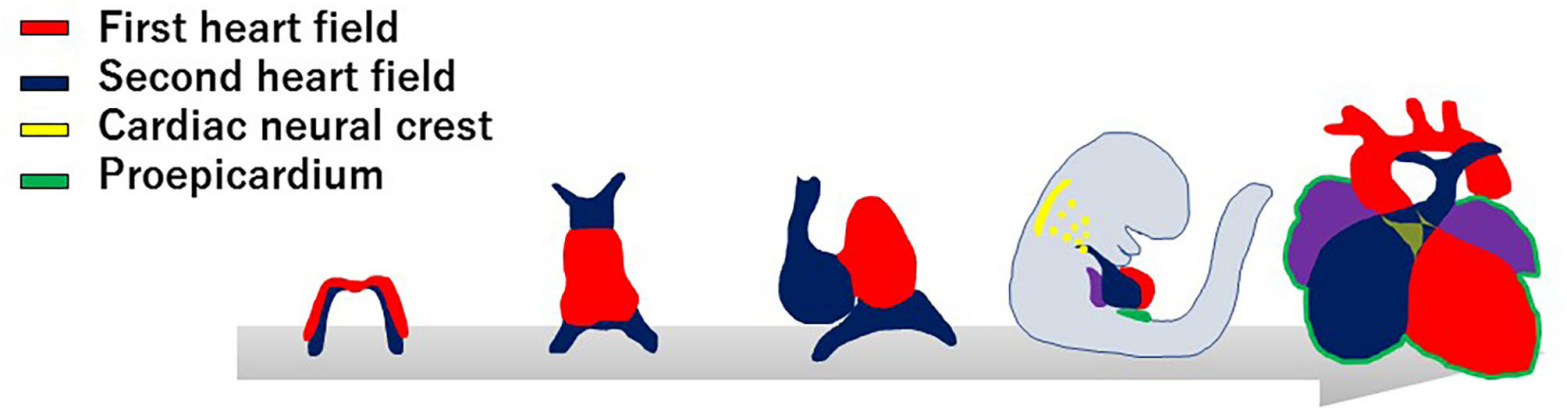
Developmental origins of the cardiovascular system. The progenitor cells of the first heart field (red) form a cardiac crescent under the anterior part of the embryo, then move ventrally to the midline and form a linear heart tube. The second heart field (blue) is situated in the medial splanchnic pharyngeal mesoderm, and migrates to the anterior and posterior parts of the linear heart tube, providing the source of the right ventricle, outflow tract, and atrial cardiomyocytes. After looping of the heart tube, cardiac neural crest cells (yellow) migrate from the dorsal neural tube to pharyngeal arch arteries III, IV, and VI, and contribute to vascular smooth muscle cells of the aortic arch and the cardiac outflow tract. At the same time, the progenitor cells from proepicardial organ (green) contact the surface of the developing heart, giving rise to the epicardium.

Current knowledge in molecular embryology suggests that the cardiovascular system is derived from at least four distinct cell lineages, namely, the first heart field (FHF), second heart field (SHF), cardiac neural crest (CNC), and proepicardial organ (PEO) (Figure 1) (6–9). The FHF stands for the crescent shaped heart primordium that is derived from the anterior lateral plate mesoderm. The FHF cells (shown in red in Figure 1) form a primitive straight heart tube, consisting of an interior endocardial layer and an exterior myocardial layer along with cardiac jelly (extracellular matrix) layer in between. In addition to the FHF, the SHF (shown in blue in Figure 1) develops medially to the cardiac crescent from the splanchnic mesoderm and lies along the pharyngeal region dorsal to the primitive heart tube derived from the FHF (10–12). Eventually, the heart tube provides a scaffold and cardiac progenitor cells derived from the SHF migrate into both anterior and posterior ends of it. The heart tube proceeds looping rightward, the cells originally from the FHF finally form exclusively the left ventricle and part of the atria, whereas cells from the SHF migrated into the anterior portions of the heart tube form a large portion of the outflow tract of and the right ventricle. In addition, cells from the SHF cross the pharyngeal mesoderm into the posterior end of the heart tube contribute to a part of the atria. Meanwhile, CNC cells (shown in yellow in Figure 1), specifically developed in the dorsal region of the neural tube between the mid-otic placode and the third somite, migrate to the outflow tract where they give rise to the outflow tract septum to separate the truncus arteriosus into the aorta and pulmonary artery (13–15). CNC cells also migrate to pharyngeal arch arteries 3, 4, and 6, where they differentiate into smooth muscle cells of the great vessels. The neural crest cells from the preotic region of the neural tube contribute to the development of coronary arteries (16). The PEO (shown in green in Figure 1) is derived from the coelomic mesothelium that overlays the liver bud and gives rise to the epicardial layer over the heart (12). Some epicardial cells invade the subepicardial space through a process of epithelial–mesenchymal transformation, and contribute to the development of the coronary vessels and connective tissues (17, 18). To further uncover the genetic architecture of CHD, it is essential to adopt an approach for identifying the specific genes involved in each of these progenitor cell lineages, and to determine how their interaction regulates cardiovascular development.

## IP_3_Rs in Cardiovascular Development

**Figure 2:**
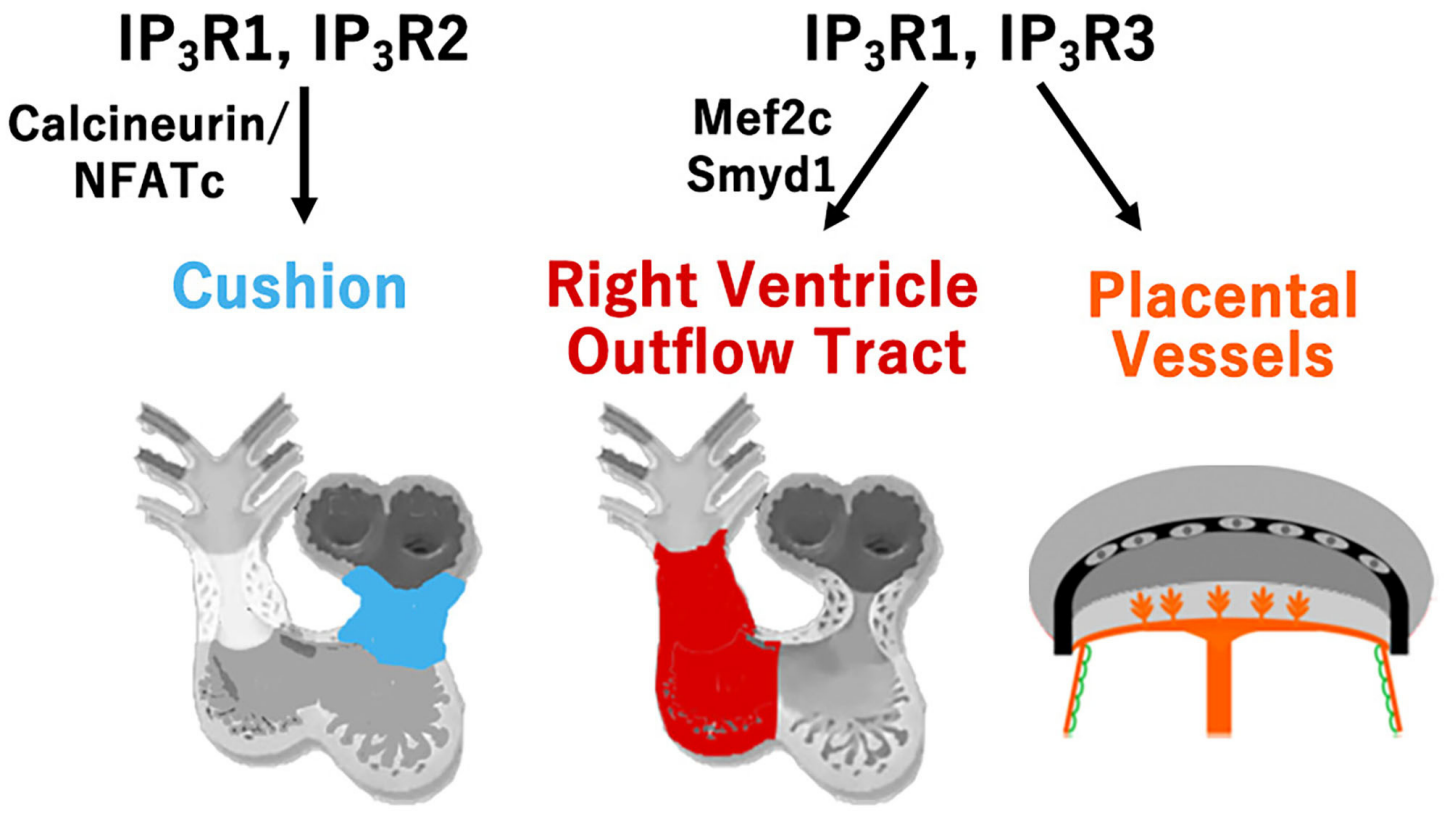
Type-specific roles of inositol trisphosphate receptors (IP_3_R) for cardiovascular development. Redundant roles of IP_3_R1 and IP_3_R2 in the development of atrioventricular cushion via calcineurin/NFATc signaling, and redundant roles of IP_3_R1 and IP_3_R3 in the development of the right ventricle and outflow tract via the Mef2c-Smyd1 pathway, and in the development of the extra-embryonic vessels of the placenta, allantois, and yolk sac are shown.

We have investigated the roles of three isoforms of IP_3_R (IP_3_R1, 2, and 3) in cardiovascular development, demonstrating their genetic redundancy (Figure 2). In particular, IP_3_R1 and 2 have redundant roles in the FHF-derived lineage, whereas IP_3_R1 and 3 exhibit redundancy in SHF-derived lineages. IP_3_Rs are intracellular Ca^2+^-release channels, which are opened by IP_3_ binding to regulate various vital processes for diverse cell functions (19). As the modifications distinguishing the isoforms vary, such as phosphorylation sites, splicing sites, and associated molecules, each IP_3_R may play a distinct role as a signaling hub offering different trajectories of cell signaling (20). In cardiovascular development, expression of IP_3_R1 was detectably higher in the atrial than in the ventricular myocardium, IP_3_R2 was mainly expressed in the trabecular layer of the ventricular myocardium, and IP_3_R3 was uniformly expressed in the atrial and ventricular myocardia from embryonic day 9.5. These dynamic and complementary expression patterns of each subtype of IP_3_R suggest their specific and/or redundant functions during the development of the heart. Although single subtype-knockout mice showed no developmental disorders and could survive after birth, IP_3_R1-IP_3_R2 double-knockout mice died *in utero* with developmental defects of the ventricular myocardium and atrioventricular canal of the heart, along with impaired Ca^2+^-dependent calcineurin/NFATc signaling by embryonic day 11.5 (21). Moreover, IP_3_R1-IP_3_R3 double-knockout embryos showed hypoplasia of the outflow tract and the right ventricle, reduced expression of specific molecular markers, and enhanced apoptosis of mesodermal cells in the SHF with reduced activity of the Mef2C-Smyd1 pathway, a transcriptional cascade essential for the SHF (22). In addition, IP_3_R1 and IP_3_R3 were found to be required for extra-embryonic vascularization in the placenta, allantois, and yolk sac at the embryonic-maternal interface (23).

## Genomic Architecture of CHD Implicated With 22q11.2 Deletion Syndrome

**Figure 3:**
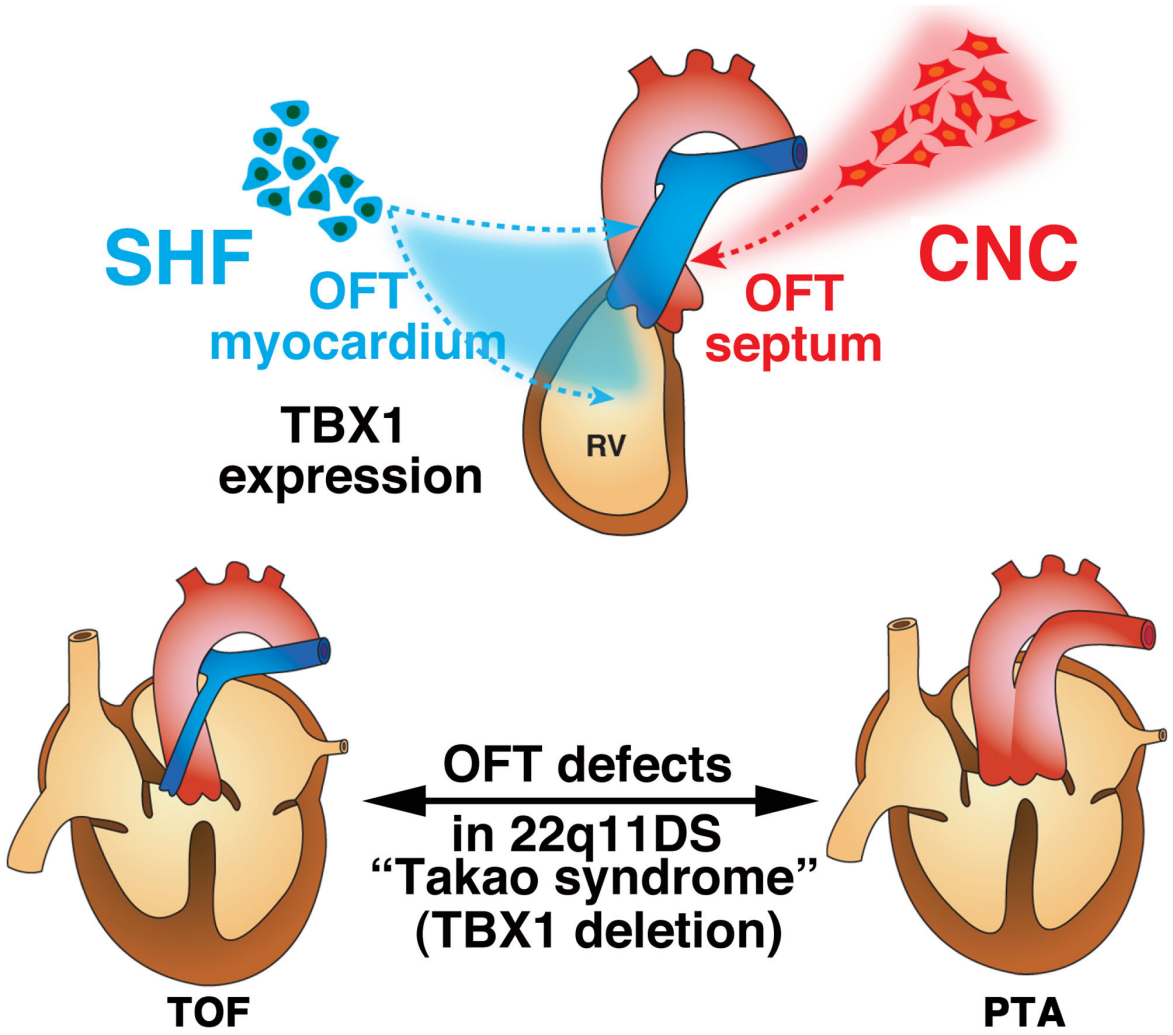
Developmental architecture underlying 22q11.2 (*TBX1*) deletion. Second heart field (SHF) cells give rise to the cardiac outflow tract (OFT) myocardium, while cardiac neural crest (CNC) cells give rise to the OFT septum during normal development. The anatomical defects in tetralogy of Fallot (TOF) and persistent truncus arteriosus (PTA) are believed to result from malrotation of the OFT and aplasia of the OFT septum, respectively. The contribution of CNC cells is thought to be essential for proper rotation and septation of the OFT. Alternatively, developmental defects of the SHF may cause hypoplasia of the pulmonary infundibulum, resulting in TOF, and a more severe decrease in the number or absence of this subset of cells may affect the development and/or migration of CNC cells, resulting in PTA. This is consistent with the notion that OFT defects ranging from TOF to PTA are highly associated with 22q11.2 deletion syndrome (22q11DS).

22q11DS is the most common chromosomal microdeletion syndrome and is also known as DiGeorge syndrome or Takao syndrome (24, 25). 22q11DS is highly associated with CHD, involving the outflow tract, including persistent truncus arteriosus (PTA) and tetralogy of Fallot (TOF). Based on observations from experimental ablation of the CNC in chicken embryos, the outflow tract defects implicated in 22q11DS were thought to be the primary defect of the CNC development that leads to the outflow tract septum of the heart. At the beginning of the twenty-first century, the transcription factor TBX1 was identified to be the major etiology of outflow tract defects in this syndrome using new genetic engineering methods to model 22q11DS in mice (26–28). Mice with null or hypomorphic mutations for *Tbx1* demonstrate PTA (28, 29). Delineation of the expression pattern of TBX1 shed further light on the molecular and cellular basis of normal and abnormal development of the outflow tract. We and other groups surprisingly revealed that TBX1 was not expressed in the CNC, but was robustly expressed in the core region of pharyngeal mesoderm in the pharyngeal arch as well as in the SHF, pharyngeal endoderm, and head mesenchyme (30–32). Moreover, we showed that TBX1-expressing descendants that represent a subset of cells originated from the SHF, predominantly contribute to the right ventricular outflow tract and pulmonary trunk (33). These findings are very intriguing because they suggest that deletion of TBX1 in 22q11DS may result in defects of CNC-derived tissues in a non-cell-autonomous fashion through the cellular interaction between CNC and the SHF. It is believed that TOF results from malalignment of the outflow tract septum, leading to an overriding aorta with malaligned ventricular septal defect (34, 35). The developmental defects of CNC is considered to cause malalignment of outflow tract septum, thus leading to TOF. Alternatively, developmental defects of the SHF may cause hypoplasia of the right ventricular outflow tract that may also result in pulmonary stenosis and malalignment of the outflow tract septum with overriding aorta (34, 35). Our data about TBX1 in the SHF provides a new insight into the developmental mechanisms underlying TOF where cellular and molecular interaction of CNC and SHF are essential (33). As for PTA in 22q11DS or TBX1 deletion, it is considered that the TBX1-expressing descendants are severely decreased in number, affecting the development and/or migration of CNC cells, thus result in complete absence of the outflow tract septum. Indeed, we recently showed that PTA in mice hypomorphic for *Tbx1* might result from agenesis of the pulmonary trunk using IP_3_R2-LacZ mice, in which a *LacZ* gene was genetically inserted in-frame at the translation initiation site of *IP*_3_*R2* locus on the mouse genome as a molecular marker (36). This developmental model is consistent of the observation that the outflow tract defects ranging from TOF to PTA are highly associated with 22q11DS (Figure 3).

## Exploring the Genomic Architecture of CHD and the Regulatory Mechanism Underlying the Interaction of Cardiac Progenitor Lineages

**Figure 4:**
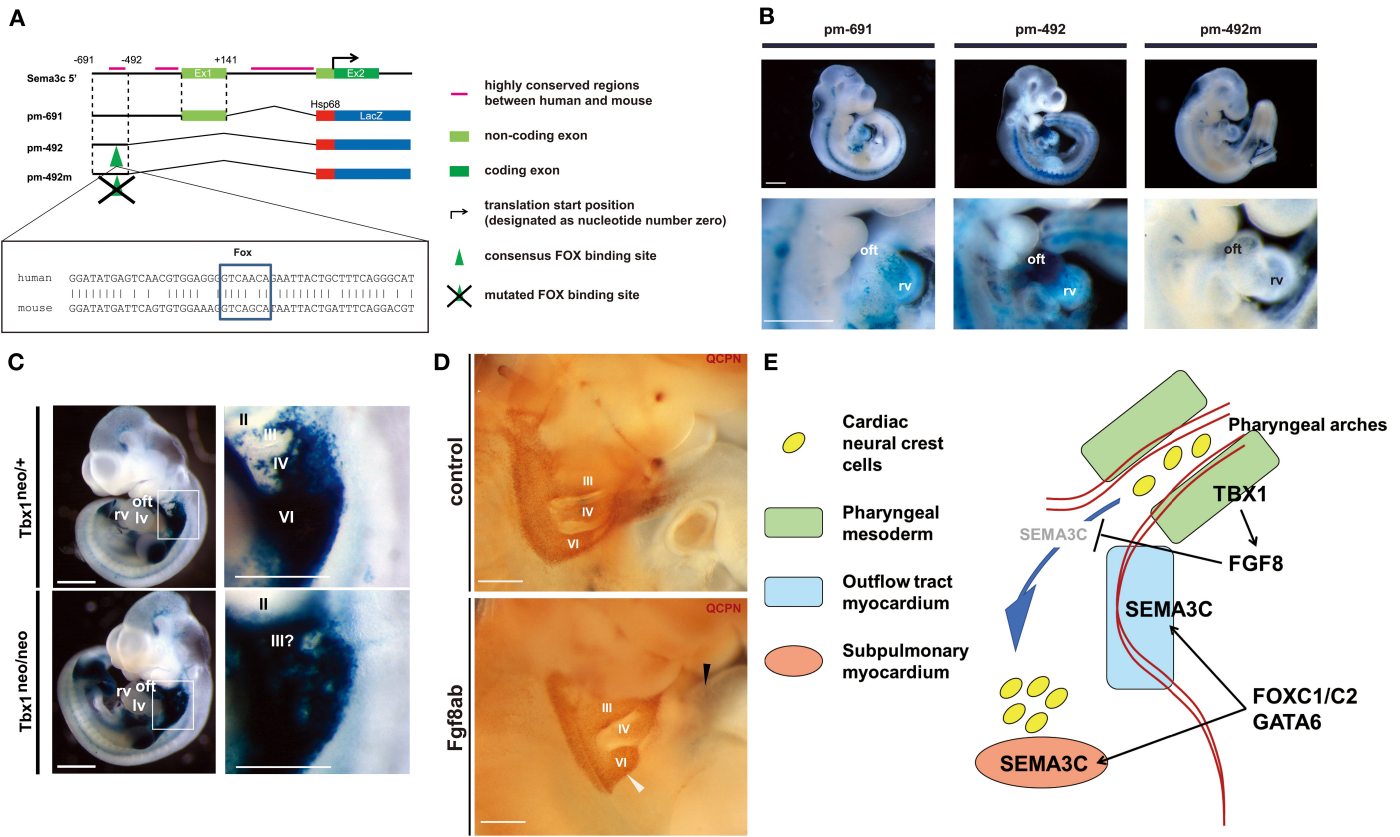
The regulatory mechanism of *SEMA3C* expression during cardiogenesis by *FOXC1, FOXC2, GATA6*, and *TBX1-FGF8* signaling. **(A)** Genomic organization of the 5′ mouse *Sema3c* locus and flanking region. Green boxes indicate exons (Ex), black bars indicate highly conserved regions between human and mouse, and the translation start site (arrow) is designated as nucleotide number zero. Each construct number is indicated on the left. The consensus Fox-binding site is indicated by a green arrowhead. The construct pm-492m has a mutated Fox site in the context of pm-492. Conserved alignments of genomic sequences including the Fox site (box) between human (upper) and mouse (lower) are shown. **(B)** Right lateral views of the hearts of representative embryos obtained with each construct. Lower lane pictures show higher-magnification views of the heart. **(C)** *LacZ* expression in *Sema3c* int1/3′-*lacZ:Tbx1neo*/+ (control: upper, left) and *Tbx1neo/neo* (Tbx1 hypomorphic: lower, left) double-transgenic (tg) embryos at embryonic day (E)10.5, reminiscent of endogenous *Sema3c* mRNA expression. High-magnification views of the pharyngeal arch region (white box) are shown in the right lane. Enhanced *lacZ* expression in the pharyngeal arch region of *Sema3c* int1/3′-l*acZ* tg:*Tbx1neo/neo* embryos (lower, right) compared to *Sema3c* int1/3′-*lacZ* tg:*Tbx1neo*/+ embryos (upper, right). **(D)** Pharyngeal arch regions of chick-quail chimeras with bilateral transplantation of cardiac neural crest cells (cNCCs). Injection of anti-Fgf8 antibody (Fgf8ab) causes less migration of cNCCs from the 6th pharyngeal arch region (white arrowhead) to the outflow tract (black arrowhead) compared to that of the control. **(E)** A working model for the regulation of SEMA3C in developing cNCCs and second heart field (SHF) progenitor cells. During normal development, TBX1 may restrict the expression of SEMA3C in the SHF along the pharyngeal arch region by blocking ectopic SEMA3C expression in cNCCs via FGF8 signaling. FOXC1, FOXC2, and GATA6 may then synergistically activate the expression of SEMA3C in the SHF during migration into the outflow tract myocardium, leading to differentiation. Panels **(A–D)** are under a Creative Commons Attribution 4.0 license.

To further elucidate the genomic architecture of CHD, we performed mutation analysis using the genome bank of Japanese patients with non-syndromic CHD, and identified *GATA6* as the genetic cause of PTA (37). Mutations in *GATA6* disturb the transcriptional regulation of downstream target genes that play an essential role in cardiac development, including semaphorin 3C (*SEMA3C*) and plexin A2 (*PLXNA2*). SEMA3C is a neurovascular guiding molecule that functions as a ligand for PLXNA2 and an attractant for CNC cells (38). Mutation of *GATA6* eliminates activation of *SEMA3C* and *PLXNA2*. Mutation of the GATA sites on the enhancer elements of *SEMA3C* and *PLXNA2* abolished these transactivation activities in the outflow tract myocardium and the CNC derivatives in the outflow tract. Further analysis of the regulatory mechanism of *SEMA3C* revealed that a molecular network involving *GATA6, FOXC1/2, TBX1, SEMA3C*, and *FGF8* plays an important role in the interaction between SHF and CNC cells (39). Moreover, we found that *TBX1* restricts the expression of *SEMA3C* to the SHF in the pharyngeal arch region by inhibiting ectopic *SEMA3C* expression in CNC cells during migration via FGF8 signaling, whereas *GATA6, FOXC1*, and *FOXC2* activate the expression of *SEMA3C* in the SHF in the outflow tract myocardium at the same time. A recent report also showed the positive regulation of *SEMA3C* expression in the proximal outflow tract by TBX1 (40). This spatial and temporal regulation of *SEMA3C* expression is essential for proper homing of CNC cells from the pharyngeal region to the outflow tract. With loss of *TBX1*, downregulation of TBX1-FGF8 signaling in the pharyngeal region may lead to misexpression of *SEMA3C* in the migrating CNC cells, resulting in the failure of their migration with ectopic aggregation, ultimately causing outflow tract defects (Figure 4) (39). Although many other genes are also associated with the regulation of CNC cell migration, our results regarding the SEMA3C regulatory mechanism provide important evidence of interactions between CNC and the SHF for the developmental basis of CHD.

## Concluding Remarks

In recent decades, detailed molecular biological analyses using genetically modified animals and accumulation of solid evidence from human mutation studies have dramatically advanced the understanding of cardiovascular development. In addition, with the recent development of stem cell science, including induced pluripotent stem cells and comprehensive expression analysis procedures using next-generation sequencing, elucidating the more detailed temporal and spatial gene regulatory mechanisms underlying cardiovascular development has become possible with evaluations at the single-cell level (9, 41–43). As a future direction for clinical application, detailed elucidation of the genomic architecture of CHD implicated in the mechanism regulating interactions between cells of multiple different origins would facilitate the development of regenerative medicine and/or preventive medicine for complex heart diseases such as CHD.

## Author Contributions

All authors listed have made a substantial, direct and intellectual contribution to the work, and approved it for publication.

## Conflict of Interest

The authors declare that the research was conducted in the absence of any commercial or financial relationships that could be construed as a potential conflict of interest.
